# Aquatic Moss Mats Are Alternative Biofilter Media for Aquaculture and Aquaponic Effluents Treating

**DOI:** 10.3390/plants15030391

**Published:** 2026-01-27

**Authors:** Irma Del Piano, Francesca Letizia, Matteo Calcagnile, Alessandro Sicuro, Laura Pecoraro, Elisa Quarta, Loredana Stabili, Tiziano Verri, Pietro Alifano, Fabrizio Barozzi, Gian Pietro Di Sansebastiano

**Affiliations:** 1 Department of Biological and Environmental Sciences and Technologies, University of Salento, Via Prov.le Lecce-Monteroni, I-73100 Lecce, Italy; irma.delpiano@unisalento.it (I.D.P.); francesca.letizia@unisalento.it (F.L.); matteo.calcagnile@unisalento.it (M.C.); alessandro.sicuro@unisalento.it (A.S.); laura.pecoraro@unisalento.it (L.P.); quartaelisa4@gmail.com (E.Q.); tiziano.verri@unisalento.it (T.V.); 2 Laboratory of Urban Farming, Department of Engineering for Innovation, University of Salento, Via Prov.le Lecce-Monteroni, I-73100 Lecce, Italy; 3 Institute of Water Research (IRSA), National Research Council (CNR), Via Roma 3, I-74123 Taranto, Italy; loredana.stabili@irsa.cnr.it; 4 National Biodiversity Future Center (NBFC), I-90133 Palermo, Italy; 5 Department of Experimental Medicine, University of Salento, Via Prov.le Lecce-Monteroni, I-73100 Lecce, Italy; pietro.alifano@unisalento.it

**Keywords:** biofilter, alternative media, aquatic moss, denitrification

## Abstract

Inert media such as plastic, ceramic or zeolite are conventionally used for wastewater biofiltration. They all need microbial activation and are essentially chosen for their surface/mass ratio, since biofiltration is entirely performed within the surface biofilm. Using biodegradable media may enhance the sustainability of the system, but it should not produce decomposition-related pollutants. Due to their surface extension, peculiar microbiota and structural resistance, aquatic moss appears to be a very good support for biofilters. Thus, in this study, we evaluated aquatic moss mats as an alternative medium for biofiltration of aquaculture or aquaponic effluents. Two moss species, *Taxiphyllum barbieri* and *Leptodictyum riparium*, were tested, also for their contribution on nitrogen metabolism and potential negative effects on hydroponic plants cultivation, due to competition for nutrients. Our proof-of-concept research demonstrates equivalence in real conditions, as inert and moss media exhibited comparable rates; however, the amount of moss required can be several times lower than that of any competing media. Preliminary results suggest the possibility to integrate moss-based biofilters in aquaculture and aquaponics technologies.

## 1. Introduction

Aquaponics is a biotechnological system combining aquaculture and hydroponics synergistically. The former is the breeding of aquatic organisms, the latter is an alternative agricultural method for the soil-less cultivation of plants: the two components are allocated in an aqueous medium from which both fish and plants take nourishment [1]. In aquaculture systems, due to feeding, fish release metabolic waste in water in the form of nitrogen compounds, which are toxic to fish at high concentrations [2]. For this reason, the activity of a biofilter in converting metabolic waste to non-toxic forms and in reducing the concentration of these compounds is crucial [3]. Biofiltration is the process commonly used to preserve water quality in aquaponics through the conversion of nitrogenous compounds, toxic to fish, into plant nutrients by beneficial microorganisms [4].

The most commonly used materials for biofilters are made of plastic products, such as Polyvinyl chloride (PVC) and Polyethylene (PE). Their use represents a potential environmental hazard because high shear pressures and friction experienced by plastic filtering media in moving bed chambers can result in the release of microplastics into the system [5]. They also require regular maintenance and need to be managed and disposed of after use [6]. Effective methods for resolving this problem include replacing plastic filtration media with natural media alternatives such as wood, shells, charcoal, coconut shells, peels and gravel [5]. Biochar and zeolite are two other biomaterials that can be used for the removal of nitrogen compounds since they have a high porosity and a large surface area that promote the adhesion of bacterial colonies [6]. In addition, plant-based wastes are widely used as support materials for microbial biofilm formation due to their advantages such as cost-effectiveness, eco-sustainability, high specific surface area and porosity, low bulk density, strong adsorption ability, high microbial population density and enhanced resistance to biodegradation as a result of their cellulose, hemicelluloses, and lignin contents. High porosity prevents the media compaction by increasing the spaces available for fluid circulation and, at the same time, the presence of pores and micropores enhances the conditions for the formation and attachment of microbial biofilm. Larger specific surface area provides more space for the growth of a microbial community, which improves the biodegradation process.

Besides environmental concerns, another important aspect to consider when selecting a biofilter is the cost of the filter materials, as commonly used biofilters can be expensive and maintenance-intensive [7]. Biomass accumulation in conventional filter media creates several negative consequences, like clogging, high flow resistance, and poor biofilm permeability, reducing biofilter performance. As an alternative, using organic materials as filter media tends to reduce the clogging problem due to the achievement of a balance between degradation of the microorganisms and the retention of solids in the filter bed under lower organic loading [8].

Recirculating Aquaculture Systems (RAS) are designed to breed fish in large numbers inside relatively small spaces where water is treated to remove waste products, toxic to fish, and then reuse it for the cultivation of plants. These systems are also referred to as integrated systems [9]. Technological research continues to explore the possibility to optimize materials and integrate more active elements in RAS configurations. In RAS, nitrogen compounds are constantly produced, converted and consumed. The nitrogen cycle is carried out in four stages: (i) nitrogen fixation, (ii) ammonification, (iii) nitrification, and (iv) denitrification by different microorganisms. An aquaponics biofilter hosts three groups of microbes: (i) organisms that oxidize ammonia to nitrite (ammonium oxidizing bacteria and archaea), (ii) organisms that oxidize nitrite to nitrate (nitrite oxidizing bacteria), and (iii) ammonia oxidizers that completely oxidize ammonia to nitrate [10]. In such a configuration, plants themselves also have the ability to metabolize nitrogen compounds, and—with their highly porous root structure and large surface area—provide an ideal environment for microbial colonization and growth, making them a valuable component for improving biological filtration efficiency [11].

Mosses are non-vascular plants that absorb nutrients and pollutants from their entire vegetative thallus, and this increases their absorption capacity in proportion to the biomass [12,13,14]. In particular, aquatic mosses live submerged in water and offer a large surface-to-weight ratio because of their ramified structure. These characteristics, along with the clumped growth, lower cost of maintenance, high removal efficiency and the regeneration of the biomass, make mosses an efficient tridimensional support for biofiltration of water [15,16].

This study evaluates two aquatic moss species, *Taxiphyllum barbieri* and *Leptodictyum riparium*, as potential alternatives to conventional biofiltration of aquaculture effluents. Their morphological and functional traits indicate a strong potential for application as natural biofilters.

## 2. Materials and Methods

### 2.1. Aquatic Moss Culture and Hydroponic Plant Growth

The aquatic moss *T. barbieri* was obtained both in axenic and semi-axenic forms from a commercial supplier (Green Greener, Policoro (MT), Italy). These mosses were grown in glass tanks used in aquaria (30 × 30 × 30 cm) and cultivated in tap water under conditions devoid of additional nutrients, with moderate aeration to ensure economical cultivation. The growth chamber was maintained at a constant temperature of 22 ± 2 °C, and the light/dark regime consisted of a 16/8 h photoperiod, with a variable light intensity, optimized at 150 µmol/m^2^·s. The growth rate of the moss was slow but steady, with a biomass increase of approx. 10% per month, despite the absence of supplementary nutrients.

Plants were grown in independent NFT Gro-Tank 1 mq (Nutriculture, Skelmersdale, UK) with a complete nutrients solution (Ca(NO_3_)_2_ 2.8 mM; KNO_3_ 6 mM; MgSO_4_·7H_2_O 2 mM); Fe-HBED 0.02 mM; (NH_4_)H_2_PO_4_ 1 mM; NH_4_NO_3_ 1 mM; KH_2_PO_4_ 1 mM; Micronutrients). Plants were placed in the NFT without any support, with nude roots.

The effect of 100 g of *T. barbieri* was tested in a 70 L medium growing 25 lettuce (*Lactuca sativa*) plants for each NFT. The experiment was set up in 3 replicas. EC was always maintained below 2500 µS by adding minimal volumes of osmotic water in all NFT to compensate evaporation.

### 2.2. Nitrogen Compounds Tests

The experiment was conducted using sterile boxes equipped with aerators, into which 500 mg of moss from each species were placed. The boxes were exposed to environmental contaminations but no microbiological inoculi were done. Aqueous solutions contained urea (3 mg/L), ammonium ion (NH_4_-N 1.4 mg/L corresponding to 5.35 mg/L of NH_4_Cl), and nitrite (NO_2_-N 0.7 mg/L corresponding to 3.45 mg/L of NaNO_2_) to simulate a RAS polluted water.

Hydrocheck Spectratest kits (Hydrocheck, Cambridge, UK) were used to measure nitrogen compounds in the aquaponic system. These kits allow, with a colorimetric method, rapid and accurate assessment of total ammonia nitrogen (TAN), nitrites (NO_2_), and nitrates (NO_3_). The concentrations were determined spectrophotometrically using a Shimadzu UV-Visible spectrophotometer accordingly to kits specification.

### 2.3. Moss-Associated Culturable Bacteria

Moss (about 10 g) belonging to the species *T. barbieri* was collected in triplicate, washed in sterile water (0.2 μm pore-filtered) to eliminate the bacteria settled on the surface, then suspended in sterile water and sonicated three times (Branson Sonifier 2200 (Brookfield, CT, USA), 60 W, 47 kHz for 1 min in an ice bath) to optimize surface bacteria detachment. The sonication was interrupted for 30 s every minute, during which time the samples were shaken manually. To enumerate culturable bacteria at 37 °C and 22 °C, 0.1 mL each sonicated sample and appropriate decimal dilutions were plated in triplicates on Bacto Plate Count Agar (PCA) (Difco, Detroit, MI, USA). After incubation for 48 h, at 37 °C and 22 °C, respectively, the growing colony forming units (CFU) were counted.

For the enumeration of culturable vibrios, 1, 5, 10 mL and serial dilutions of each sample (moss homogenate) were filtered in triplicate on 0.45 μm Millipore pore size filters that were aseptically placed onto thiosulphate-citrate-bile-salt-agar (TCBS) (Difco, Detroit, MI, USA) plus 2% NaCl. After incubation, for 48 h at 22 °C and 35 °C, the colonies of presumptive vibrios (yellow or green), grown on TCBS agar, were counted and reported as CFU/mL [17]. The incubation temperature of 35 °C was chosen to estimate the fraction of vibrios potentially pathogenic to humans. The lowest incubation temperature (20–25 °C) was selected to detect some Vibrio spp., including V. anguillarum, that do not thrive at 37 °C [18].

In addition, pieces of each moss sample were gently washed in sterile water (0.2 μm pore filtered) to eliminate the bacteria settled on the surfaces, then directly placed on PCA agar and TCBS plates, respectively, and incubated at 22 °C, 37 °C for PCA and 22 °C and 35 °C for TCBS for 48 h.

### 2.4. Moss-Associated Microbiota: 16S rRNA Metabarcoding

About 5 mg of each moss sample was collected, rinsed three times in sterile water, and subsequently used for DNA extraction using the E.Z.N.A.^®^ Soil DNA Kit (D5625, Omega Bio-tek, Norcross, GA, USA), following the manufacturer’s instructions. The extracted DNA was assessed for quality and quantity using UV spectrophotometry (NanoDrop^®^ ND-1000, Thermo Fisher Scientific, Waltham, MA, USA) and agarose gel electrophoresis (1.5%, 75 V). After extraction, the DNA samples were sent to Genomix4life S.R.L. (Baronissi, Salerno, Italy) for quality control, next-generation sequencing, and preliminary bioinformatics analysis. The sequencing and bioinformatics procedures were conducted as previously described [19,20]. α-diversity analysis was performed using the Python 3 package scikit-bio.

### 2.5. Use of Fish in the Aquaculture System

The land-based assisted production macrosystem in aquaculture in the Urban Farming Lab at University of Salento was equipped with an experimentally designed biofilter divided into two distinct and independent compartments. The first compartment operated through a traditional biofiltration system with a filter bed activated with a commercial bacterial activator, while the second relied on an aquatic moss biofilter. Their experimental design is described in detail in the Supplementary File. Each compartment consisted of a fish production tank, a settling tank, three biofiltration tanks, two supplemental floating plant tanks (not relevant for this experiment but described in the Supplementary File), a process water storage and recirculation tank, a recirculation pump, a UV sterilizer, and a piping system that served the entire facility.

Fish used in the experiment consisted of a lot of 150 Apulian naturalized Nile tilapia (*Oreochromis niloticus*) [21], obtained from Azienda Ittica Agricola Residence San Nazario srls (Lesina, Foggia, Italy). The initial size of 90 days old fishes was measured before the experiment, and out of 75 selected uniform-sized fish (average body weight: 41.5 ± 8.3 g) (means ± SD; n = 75) 2 groups were randomly composed and distributed into 2 tanks (37–38 fish each tank). In detail, fish were acclimatized under laboratory conditions (water temperature: 24 ± 1 °C; photoperiod: 12 h light/12 h dark) for 2 weeks in tanks (tank volume: 2000 L each) containing constantly aerated water (water volume: 1800 L each). During this period, the fish were fed with a complete commercial diet (Veronesi CFW 4; extruded pellets, 4 mm diameter). The diet composition was: 35% crude protein, 10% crude fat and crude fiber 4.80%, ash 5.95%, calcium 0.60%, phosphorus 0.80%, sodium 0.12%.

The study was carried out between March 2024 and May 2024 for 3 months in experimental tanks at the University of Salento.

The fish were fed to satiation by hand twice a day, 7 days per week. Temperature and dissolved oxygen were determined daily in the morning and in the afternoon with a digital oximeter (YSI 55 Hexis). At 30, 60 and 90 days from the beginning of the experiment, three individuals from each tank were singularly collected, anesthetized (0.3% MS-222, 300 mg/L, Sigma-Aldrich, Saint Louis, MO, USA) and weighted by an Analytical Balance Cubis^®^ MSA SARTORIUS (readability 0.01 mg, Göttingen, Germany) for growth measurement and biomass gain evaluation.

At the end of the experimentation trial, survival rate (%), biomass growth, specific growth rate, and coefficient of variation for length were evaluated using the following equations: Survival rate (%) = (number of fish at the end/number of fish at the beginning) × 100; Biomass gain (g) = final individual weight − initial individual weight; Specific growth rate (%) = (ln final weight − ln initial weight) × 100/feeding days, K = (W/Lcm^3^) × 100.

### 2.6. Statistical Analysis

Statistical analysis and graphic rendering of the result were produced by using R Core Team (V. 4.5.1) and R Studio Team software (V. 2026.01.0 Build 392). Statistical analysis by a Kruskal–Wallis test with a post hoc test using Fisher’s least significant difference and a *p* value of 0.05 was applied to quantitative value of nitrogen compounds and solution pH variation, plant growth parameters and pigments contents. The Bonferroni multiple comparison test was used for adjusting the *p* values. The n value of 3 corresponds to 3 independent experiments with 3 (nitrogen compounds and solution pH variation) or more (plant growth parameters and pigments contents) replicas.

Statistical analysis by Student’s *t*-test with a *p* value of 0.05 was applied to fish (Control vs. MOSS) growth parameters.

## 3. Results

### 3.1. Structure of the Moss Mass and Active Surface Area

The aquatic mosses considered in this study are distinguishable by the shape of their leaves and leaves insertion on the stem, but their size and density are similar [16]. Starting from the length of the leaflets, equal to 2.5–3 mm, and considering they are formed by a single cells layer, a general estimation of surface corresponding to weight was possible. We considered that 1 mm gametophyte filament hosted 2 leaflets and offered a minimal surface of about 5 mm^2^. We did not consider the internal surface of dead cells. Scaling up the estimation, 1 g of moss biomass offers a minimum of 80 m^2^ of active surface. A single cubic meter of biofilter can accommodate 125 kg of vital moss with an estimated active surface area of 10.000.000 m^2^/m^3^.

### 3.2. Moss Metabolization of Nitrogen Compounds

The ability of the two moss species, *T. barbieri* and *L. riparium*, to absorb nitrogen compounds was assessed. The nitrite and ammonium concentrations used in this study were selected based on the limit concentrations reported in the literature [22] and considering the natural fluctuations of nitrogen compounds typically observed in a RAS [23,24].

**Figure 1:**
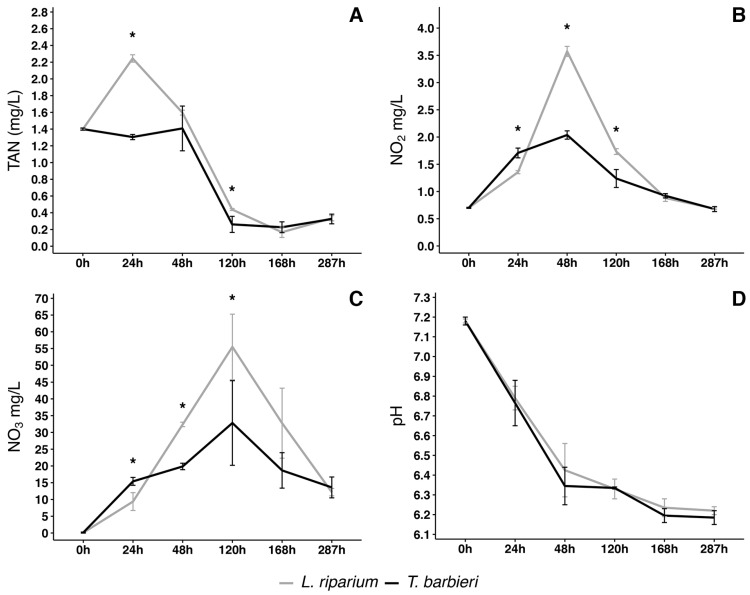
Changes in nitrogen compounds and pH in solutions treated with moss for 287 h. (**A**) Total Ammoniacal Nitrogen (TAN), (**B**) nitrite (NO_2_), (**C**) nitrate (NO_3_) variation of concentration in time, and (**D**) pH variation in time in *L. riparium* and *T. barbieri*. Statistical analysis performed by Kruskal–Wallis test with a post hoc test using Fisher’s least significant difference and a *p* value of 0.05. “Bonferroni” was used for adjusting the *p* values. Asterisks indicate significative difference in the value between the two mosses at the specific time point. Vertical bars show standard deviation. *n* = 3.

Both moss species exhibited a similar behavior in metabolizing Total Ammoniacal Nitrogen (TAN), although the concentration trend appeared more linear with *T. barbieri* (Figure 1A). With *L. riparium*, TAN concentration rapidly increased to a peak within 24 h, likely due to the decomposition of urea in the water, resulting in ammonium production. After this initial period, TAN concentration began to decrease and, after 48 h, it decreased very rapidly for both moss species, reaching a minimum concentration at 168 h.

The trend in nitrite concentration (Figure 1B) is correlated with the trend in ammonium concentration (Figure 1A). Specifically, for *T. barbieri*, the nitrite concentration reached an initial peak at 24 h, coinciding with a slight decrease in ammonium concentration. Instead, in the solution treated with *L. riparium*, the nitrite concentration reached its maximum at 48 h, which corresponded to a decrease in ammonium concentration. The increase in nitrite concentration is due to the oxidation of ammonium to nitrite, in accordance with the first step of the nitrification process showing that moss metabolism may somehow catalyze such step or it is integrated already by nitrifying bacteria. After 48 h, nitrite concentration decreased rapidly for both mosses, reaching a minimum after 120 h.

The concentration of nitrate significantly escalated, reaching its peak at 120 h (Figure 1C). This corresponds to the minimum in the nitrite concentration trend, in agreement with the progression of the nitrification process, during which nitrite is oxidized to nitrate. Following this 120 h period, the resultant nitrate was possibly assimilated by the moss.

During the experimental period of 287 h, the pH of the solutions was measured daily. A decrease over time was observed, essentially equivalent with the two moss species and essentially due to acidification induced by moss metabolism (Figure 1D).

### 3.3. Microbiology of the Aquatic Moss

**Figure 2:**
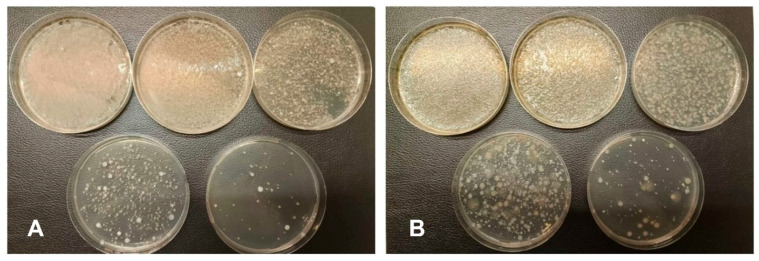
Petri dishes with Plate Count Agar indicating the growth of moss-associated culturable bacteria at 37 °C (**A**) and 22 °C (**B**).

**Figure 3:**
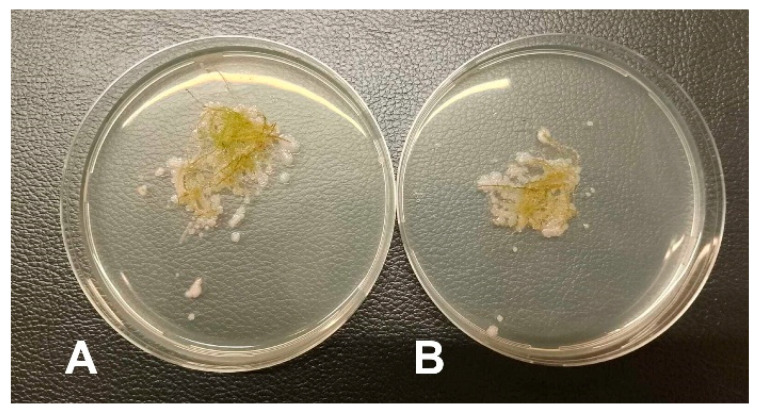
Bacteria grown from the surface of moss after incubation in a Petri dish containing PCA agar. Note the growth of a white coating on the plates due to the growth of moss-associated culturable bacteria at 37 °C (**A**) and 22 °C (**B**).

*T. barbieri* was grown under low bacterial load conditions using deionized water and without salt and carbon sources for a long time (3 years). Before the experiments in which the moss was used as a biofilter in a real RAS, approx. 50 mg of biomass from each moss type was collected to analyze the moss-associated microbiota using 16S rRNA metabarcoding method. Additionally, bacterial counting methods were used to calculate the number of moss-associated culturable bacteria. In particular, culturable bacteria at 37 °C were 5.97 × 10^6^ CFU/mL (Figure 2A) and those at 22 °C were 10 × 10^6^ CFU/mL (Figure 2B). By contrast no vibrios grew on TCBS agar. Furthermore, when moss pieces were placed directly on PCA agar plates at 22 °C and 37 °C from a macroscopic point of view, after 48 h incubation, we observed the growth of a coating around the moss due to the growth of moss-associated culturable bacteria at 37 °C (Figure 3A) as at 22 °C (Figure 3B). In contrast, no coating was observed when the same procedure was performed on TCBs plates.

The data obtained from 16S rRNA metabarcoding were analyzed to assess the diversity of the moss-associated microbiota and to characterize its taxonomic composition at the phylum and family levels.

The α-diversity of the microbiota of the two moss samples was analyzed at the family and phylum levels using 3 diversity indices: Chao Index, Shannon Index, and Berger-Parker Dominance (Table 1). First, according to the Chao Index, *L. riparium* showed slightly higher richness (281.93) than *T. barbieri* (263.67) at the family level. In contrast, at the phylum level, both mosses exhibited similar values (Chao Index 39.2 for *L. riparium* and and 39.0 for *T. barbieri*). According to the Shannon Index, which measures diversity by considering species number and distribution, *T. barbieri* and *L. riparium* exhibited similar values at the family and phylum levels, indicating comparable diversity. Finally, based on the Berger-Parker Index, which quantifies the relative dominance of the most abundant species, *T. barbieri* had a higher value at both the family level (0.21) and the phylum level (0.51) compared to *L. riparium* (0.18 and 0.46, respectively). This suggests that *T. barbieri* harbors a greater dominance of the most abundant species than *L. riparium*. Both microbial communities exhibited high diversity and evenness, with minimal differences between *T. barbieri* and *L. riparium*. However, *T. barbieri* showed a greater estimated richness and a higher number of families, while *L. riparium* appeared to have a slightly more balanced community with less dominance of a single species. Overall, high microbial diversity can be observed despite the fact that the mosses have been treated to remove surface-associated bacteria and grown under artificial and partially sterile conditions.

**Figure 4:**
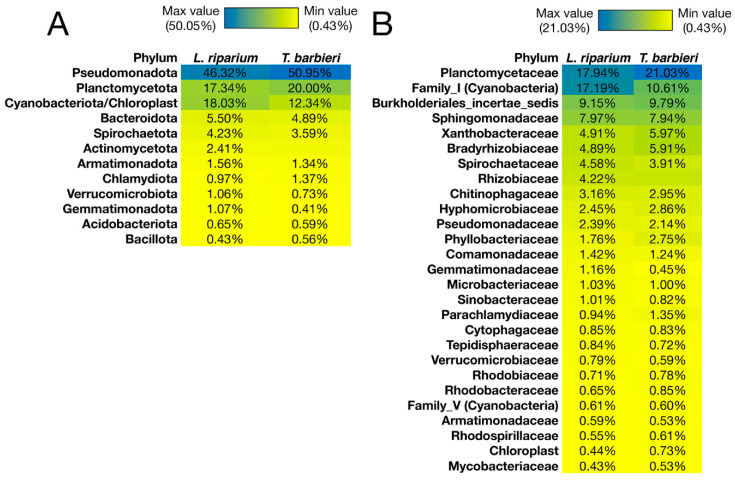
Composition of the microbiota in the two moss species at the taxonomic level of phyla (**A**) and families (**B**).

From a structural point of view, the microbiota of *L. riparium* and *T. barbieri* was dominated by three phyla (Pseudomonadota, Cyanobacteriota/Chloroplast, and Planctomycetota), which collectively reached a relative abundance greater than 80% in both mosses (Figure 4; and Supplementary File). Planctomycetaceae was the most abundant family in both samples, with relative abundance of 21% in *T. barbieri* and 17.9% in *L. riparium* (Figure 4). In the microbiota of *L. riparium*, Family_I, belonging to the phylum Cyanobacteria, showed a relative abundance similar to that of Planctomycetaceae (17.2%), while in *T. barbieri* the Family I showed a relative abundance of 10.6% (Figure 4). Among other families, 6 were slightly prevalent in *T. barbieri* (Rhizobiaceae, Parachlamydiaceae, Hyphomicrobiaceae, Phyllobacteriaceae, Bradyrhizobiaceae, and Xanthobacteraceae), and 7 were slightly prevalent in *L. riparium* (Gemmatimonadaceae, Spirochaetaceae, Pseudomonadaceae, Chitinophagaceae, Sinobacteraceae, Comamonadaceae, and Microbacteriaceae) (Figure 4).

### 3.4. Comparison of Inert and Moss-Based Biofiltration in Nile Tilapia Cultivation

To evaluate the ability of moss to absorb the nitrogenous compounds released by fishes in a real RAS, an experiment was set to compare biofiltration efficiency of a biofilter based on inert material activated with a complete commercial bacterial activator (dose 5 mL/40 L Stability, Seachem, Madison, GA, USA) pre-incubated for one week to a biofilter with the same volume but containing a fixed amount of moss. To define the amount of moss biomass to be used, we considered the results in Figure 1 despite a direct calculation of equivalence, which was impossible due to the very different operating conditions.

We estimated that a moss-based filter eliminates approximately 30 mg/h of NO_2_ per kg, slowing down efficiency only after eliminating 1.35 g/kg. At the same time, the same moss can accumulate from 2 to 274 mg/h of NO_3_ per kg, slowing down efficiency after the accumulation of a total of 110 mg/kg. Despite the possible slowdown due to the saturation of the system, we hypothesize that the biofilter remains active even in conditions of equilibrium of the system.

**Figure 5:**
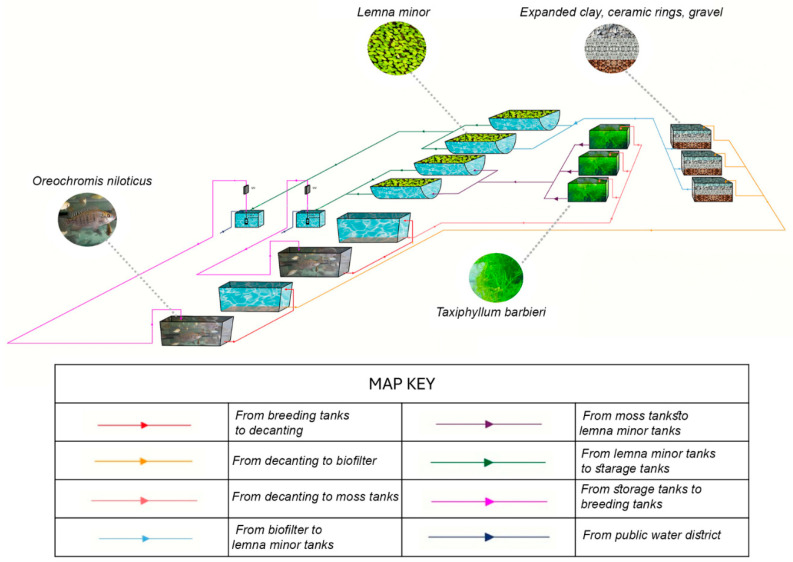
Diagram of the experimental RAS.

**Figure 6:**
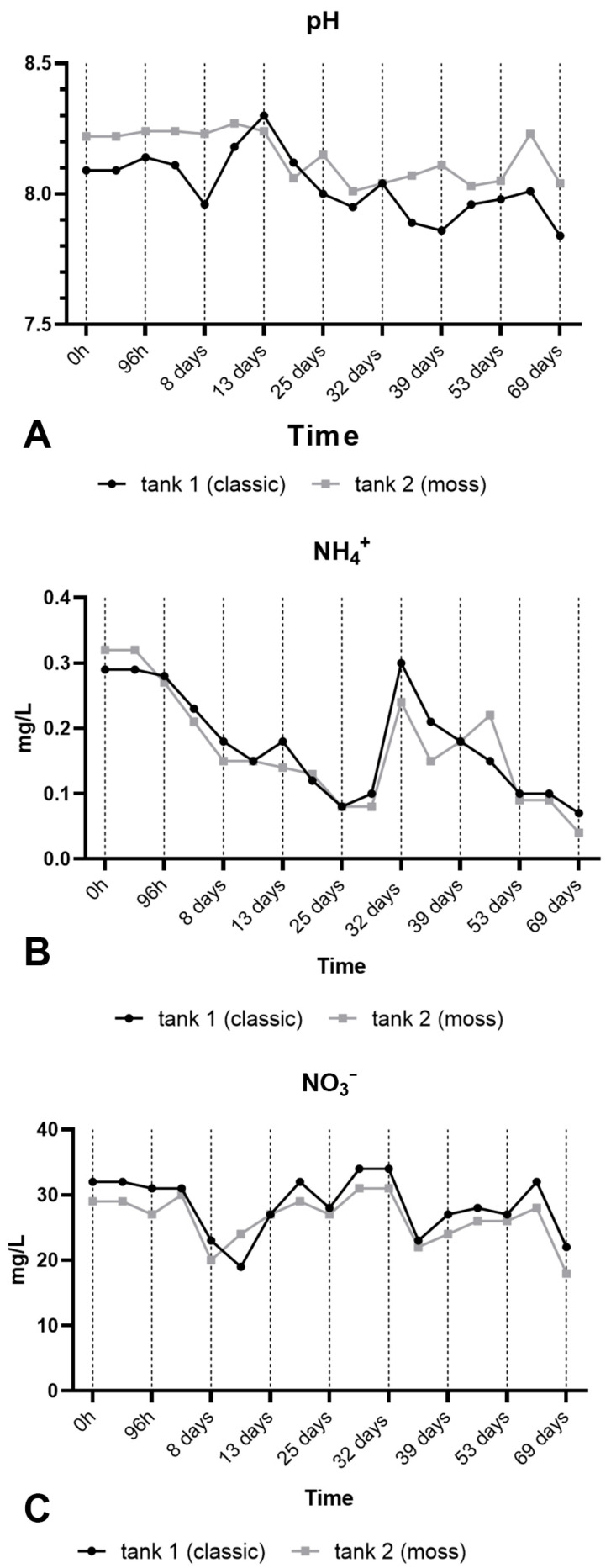
Values monitored in the two filters over a period of three months. (**A**) pH, (**B**) NH_4_^+^, (**C**) NO_3_^−^.

The study was performed in a land-based assisted production macrosystem in aquaculture containing Nile tilapia (see Materials and Methods and Supplementary File for details). A diagram of the RAS is shown in Figure 5 and will be further described in detail in Supplementary File. During the experiment, the following parameters were measured: survival rate (%), biomass gain, specific growth rate (SGR), and condition factor (K). Fish fed in either the control tank or the aquatic moss tank (MOSS) showed 100% survival in both groups. At the end of the experiment, the biomass gain was 81.47 g for the Control group and 74.27 g for the MOSS group. The SGR was slightly higher in the Control group (0.83%) but comparable to the MOSS group (0.78%). Conversely, the K index was higher in the MOSS group (2.39) than in the Control group (2.10). However, the observed differences in all analyzed parameters were not significant (*p* value > 0.05). As shown in Figure 6, monitoring of pH (A), NH_4_^+^ (B), NO_3_ (C) values showed the substantial equivalence of performance of the two filters over a period of three months.

### 3.5. Moss Effect on NFT Hydroponic Lettuce Cultivation

**Figure 7:**
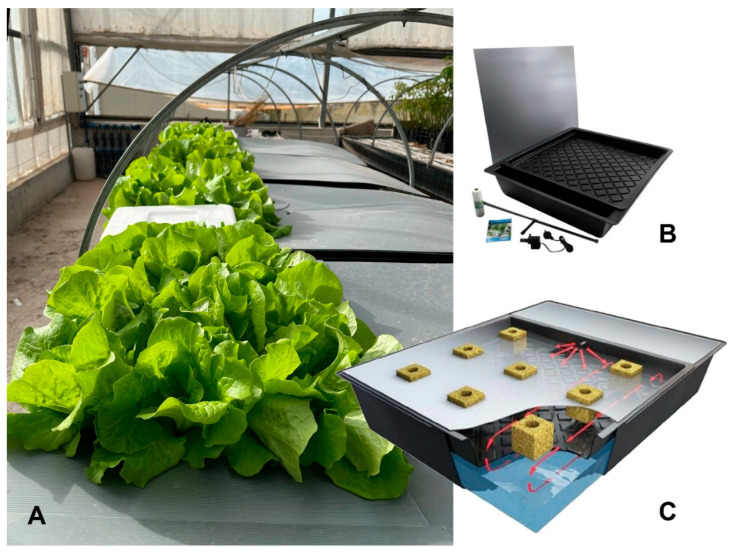
Plants were grown in independent NFT tanks as shown. (**A**) Plants in independent tanks; (**B**) Gro-Tank 1 mq complete kit; (**C**) Gro-Tank re-circulation scheme.

**Figure 8:**
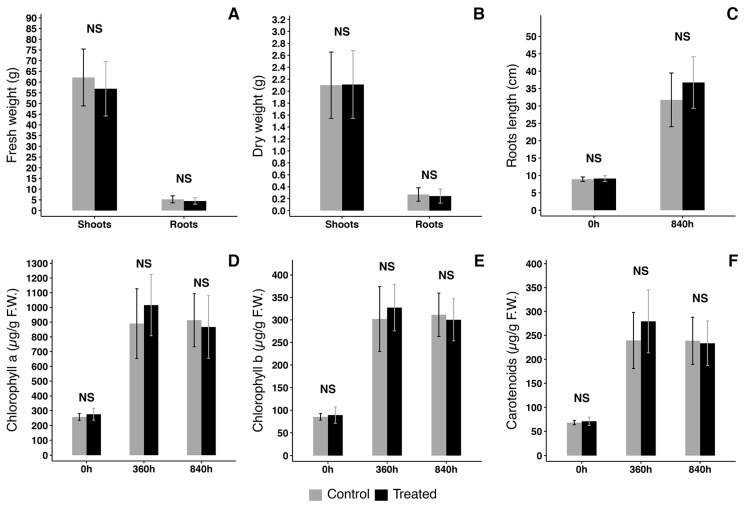
Characterization of plants grown on NFT in control conditions or co-cultivated with moss T.barbieri. (**A**) Fresh weight comparison; (**B**) dry weight comparison; (**C**) roots length comparison; (**D**) Chlorophyll a (Chl a) quantity comparison at 3 growth intervals; (**E**) Chlorophyll b (Chl b) quantity comparison at 3 growth intervals; (**F**) carotenoids concentration comparison at 3 growth intervals. Statistical analysis performed by Kruskal–Wallis test with a post hoc test using Fisher’s least significant difference and a *p* value of 0.05. “Bonferroni” was used for adjusting the *p* value. NS = non-statistically significant difference. Vertical bars show standard deviation. n = 3.

An aquaponic RAS is integrated with fish growth and hydroponic plant cultivation, so we investigated the effect of 100 g of moss in a 70 L NFT hydroponic system growing lettuce (Figure 7). Parameters were monitored and pH remained between 5.5 and 6.5. Figure 8 reports some of the morphometric data of plants after growth in control conditions or in presence of moss (treated): fresh weight (Figure 8A), dry weight (Figure 8B) and roots length (Figure 8C). No significant anatomical or fitness differences were detected. A trend for the development of longer roots in treated plants had no statistical significance. Further fitness analysis was performed measuring total amounts of Chlorophyll a (Figure 8D), Chlorophyll b (Figure 8E) and carotenoids (Figure 8F). Again, even if a trend for a larger production of carotenoids could be observed in treated plants, no statistically significant differences were detected.

## 4. Discussion

The aquatic mosses considered in this study belong to two different families, Amblystegiaceae and Hypnaceae, but share similarities that make them potentially useful for wastewater biofiltration. *T. barbieri* (Hypnaceae), also known as “Java moss” is a native species of South East Asia and is known to grow in all types of water and adapt very easily to even extreme chemical-physical conditions. It is a moss acrocarpous with sympodial growth and its color ranges from dark green to light yellow; it forms dense cushions with irregular branching. *L. riparium* (Amblystegiaceae), also known as “Stringy moss”, is widespread almost all over the world (except for Australia and Pacific islands). It is a pleurocarpous moss with monopodial growth; its yellow-olive gametophyte forms mats with irregular ramifications and appears very branched.

The two moss species are distinguishable by the characteristics of the leaves. As for their insertion on the stem, in *L. riparium* the leaves are inserted transversely and are spreading, with the tips pointing outwards forming a 90° angle respect to the stem; therefore, the general appearance of the leaf structure is flattened. In *T. barbieri,* leaves are ectopatent, forming an angle less than 45° with respect to the stem. As for the shape, in *L. riparium* the leaves (2.5–3 mm) are oblong-lanceolate with acuminate apex and they are also decurrent. The leaves of *T. barbieri* (2.5–3 mm) instead, are oval-lanceolate briefly acuminate with margins slightly denticulate and not decurrent at the base [16]. A general estimation of surface corresponding to weight was possible, considering that 1 mm gametophyte filament hosted 2 leaflets, offering a minimal surface of about 5 mm^2^, not considering the internal surface of cells after their death. Starting from length measure, it was possible to scale the estimation and indicate that 1 g of moss biomass offers a minimum of 80 m^2^ of active surface. A single cubic meter of biofilter can accommodate 125 kg of vital moss with an estimated active surface area of 10,000,000 m^2^/m^3^. Thanks to moss mats characteristics, we can compare active surface area with that of other biofilter supports, considered among those with highest active surface area, such as bioelements in polypropylene with 300–859 m^2^/m^3^ [25,26]. Even considering that mosses will need light exposure, it is easy to estimate that moss based biofilters may be designed to easily meet the performances obtained with other technological solutions and eventually improve them.

Fish release in water mainly ammonia, which is highly toxic, along with small amounts of urea, which is much less harmful. This last one, according to the nitrogen cycle, is converted to ammonia: in particular, it is partially hydrolyzed to ammonium carbamate and subsequently decomposed to produce ammonia and carbon dioxide [27]. Ammonia, due to its physicochemical properties, is mainly found in water as ammonium (NH_4_^+^) and this depends on pH, temperature and salinity [28]. Total ammonia nitrogen (TAN) consists of the sum of the dissociated form (ammonium ion NH_4_^+^) and undissociated form (ammonia, NH_3_) [29].

Nitrification is the sequential oxidation of ammonia to nitrate in two steps. The first step involves the oxidation of ammonia to nitrite mediated by Nitrosomonas; the second step continues with the oxidation of nitrite to nitrate by Nitrobacter [30].

The ability of two moss species, *T. barbieri* and *L. riparium*, to absorb nitrogen compounds was assessed. The experiment was conducted using sterile boxes equipped with aerators, into which 500 mg of moss from each species were placed. These moss samples were exposed to aqueous solutions containing urea (3 mg/mL), ammonium ion (5.35 mg/mL), and nitrite (3.45 mg/mL) to simulate a RAS polluted water. The two moss species exhibited similar behavior, suggesting that the choice of one species over the other could depend on the preference for native species to avoid potential ecological issues related to the introduction of non-native organisms. During the experiment, periodic measurements of total ammoniacal nitrogen, nitrite, and nitrate concentrations were taken, and their dynamics were found to align with the typical metabolic processes of their transformation.

Total Ammoniacal Nitrogen (TAN) concentration rapidly increases to a peak within 24 h, likely due to the decomposition of urea in the water, resulting in ammonium production. After this initial period, TAN concentration decreases. Ammonium is presumably oxidized to nitrites, which concentration increases, reaching a peak between 24–48 h, which corresponds to the decrease in ammonium concentration. After 48 h, nitrite concentration decreases for both mosses. This may be surprising when the axenic set-up of the experiment is considered. The possibility that moss metabolism may somehow catalyze such step has to be investigated further but processes in the incubation boxes may be also integrated by nitrifying bacteria rapidly proliferating on the moss surface. In agreement with the progression of the nitrification process, nitrite is oxidized to nitrate. Following the 120 h period of increase, nitrate concentration drops down, possibly because the compounds are assimilated by the moss. During the experiments, the pH values stabilize to an optimal for plants in hydroponic cultivation. As shown in Figure 1D, moss certainly contribute to this stabilization but the lack of relevant differences in the experiments shown in Figure 6 A, suggest that the contribution is limited.

Over the years, the pursuit of alternative biofilter media to use in RAS has been driven by two goals: improving the efficiency of the biological filtration process while simultaneously reducing the overall cost associated with their maintenance and disposal [5]. Both aquatic moss species appear to be promising filtering material thanks to their gametophyte properties. In fact, moss gametophyte forms dense mats on the organism’s integrity and vitality. They have a very strong and resistant thallus that can be handled and compressed with no significant damage, not releasing debris and being compacted to fit filtering containers of different volume and shape. This can represent a valid mechanical filter for particles resuspended in water and can also work as an optimal support for the growth of nitrogen-cycling bacteria.

This is supported by the results from the microbiological analyses revealing the presence of moss-associated culturable bacteria and corroborated from the metabarcoding results indicating that the microbial communities of both mosses are extremely rich in bacteria that participate in the nitrogen cycle. First, the family of Plactomycetaceae includes several species known as anaerobic ammonium oxidation (Anammox) bacteria. The anammox process involves the conversion of ammonium (NH_4_^+^) and nitrite (NO_2_^−^) to nitrogen (N_2_), which is then released into the atmosphere [31]. In addition, this bacterial family is known to form biofilms on macroalgae [32,33], facilitating the uptake of dissolved organic nitrogen [32]. On the other hand, many bacterial families (Family_I, Singingomonadaceaceae, Xanthobacteraceae, Bradyrhizobiaceae, Spirochaetaceae, Rhizizobiaceae, Hyphomicrobiaceae, Pseudomonadaceacae, Comamonadaceae) include nitrogen-fixing species [34,35,36,37,38,39,40,41,42,43]. Planctomycetes have also been linked to the nitrogen fixation process on the ocean surface [44]. Some of these bacterial families are also involved in the process of denitrification, such as Pseudomonadaceae and Bradyrhizobiaceae [36,45]. Other bacterial families associated with the denitrification process, such as Micrococcaceae, Alcaligenaceae, Rhodocyclaceae were detected in the microbial communities. However, these families show a low relative abundance (<1%). Finally, bacterial families, such as Xanthobacteraceae, Chitinophagaceae, Hyphomicrobiaceae, Comamonadaceae include bacterial species involved in the nitrification process or that have been associated with ammonia oxidation or consumption [41,46,47]. Other bacterial families involved in nitrification, such as Nitrosomonadaceae and Nitrospiraceae, have been detected in mosses with a low relative abundance. Overall, these data show that the moss microbiota is dominated by bacteria that participate in the nitrogen cycle, particularly nitrogen-fixing bacteria.

The reported data are consistent with other studies on terrestrial and aquatic mosses, which highlight the importance of moss-associated microbiota in the nitrogen cycle and nitrogen fixation [48,49,50,51,52,53,54]. The main difference observed is the relative abundance of bacteria belonging to the phylum Planctomycetota, which in this study is 17% in *L. riparium* and 20% in *T. barbieri*, while in other moss species, the abundance of this phylum is lower, ranging from 9% to 4% [48,49,52].

We investigated the aquatic moss performance in a RAS to explore its potential as a natural and eco-sustainable substitute for the artificial materials traditionally used in biofiltration [14,55]. In a RAS, the primary input of nitrogen is via proteins in fish feed as Norg. These are ingested, metabolized and transformed by the fish into ammonia (NH_3_), which is mainly released into the water via passive gill diffusion. The remaining organic nitrogen (Norg) present in the fish excreta, non-consumed feed and decaying biomass is mineralized to NH_4_^+^ [56]. Total ammoniacal nitrogen (TAN) is the sum of the dissociated form (ammonium ion, NH_4_^+^) that is relatively non-toxic to fish and the undissociated form (ammonia, NH_3_) that is highly toxic [22]. These inorganic nitrogen forms (Ninorg) can be further transformed to Nitrite (NO_2_) and Nitrate (NO_3_) via nitrification, to nitrogen gas via denitrification and/or anaerobic ammonium oxidation, or assimilated into biomass by microbes and plants [56]. When the level of ammonia in water increases, the rate of excretion in fish decreases. This causes an increase in the level of ammonia in blood and tissues. As a result, the pH of blood increases and causes adverse metabolic effects on enzyme-catalysed reactions and membrane stability. It also reduces the oxygen consumption of tissues, damages gills and reduces the blood ability to carry oxygen [57].

NO_2_ is an intermediate product of the conversion of ammonia into nitrate, toxic to aquatic organisms. However, it is a very unstable compound and is easily oxidized to nitrate in the presence of oxygen or reduced to ammonia under anoxic conditions. Nitrite in fish reacts with hemoglobin to form meta-hemoglobin, which impairs oxygen transport capacity, causing hypoxia and cyanosis which in turn lead to stress and consequent mortality. NO_3_ is the final product of the nitrification process and is considered to be relatively less toxic to fish [58].

The fish biomass gain was slightly higher in the RAS running with a classic biofilter but comparable to the gain in the RAS running with the moss-based biofilters. Conversely, the K index was higher in the last than in the control conditions. However, the observed differences in all analyzed parameters were not significant (*p* value > 0.05), showing that the moss-based biofilters had no evident influence on the fish growth confirming that the use of moss is not detrimental to fish growth.

The influence on plant growth was tested independently to reduce the variability on nutrients availability. The need for nitrogenous compounds for plant nutrition in an aquaponics system suggests that the ratio of 1 kg moss/45 mg NO_2_, can be lowered according to the needs of the hydroponic culture. NO_3_ instead seem to saturate the system and therefore remain available to the plants. Salts are typically the limiting factors with iron being the most critical nutrient. Performing experiments in closed NFT we had full control of salts available. Analyzing the morphometric parameters of fresh and dry weight and of root length, in parallel with physiological analysis of chlorophyll a and b and of carotenoids, we can have small indication of an ongoing biological competition (showed for example by longer root growth) but no significant detrimental effect can be evidenced.

## 5. Conclusions

Our data demonstrates effectiveness of moss media as biofiltering material showing that it is possible to estimate a quantity able to perform similar action as other tridimensional supports for biofiltration. While acting as biofilter, moss biomasses have no detrimental effects on both fish or plants growth. Advantages emerging from our characterization are: the reduced quantity of moss required, the direct influence on associated microbiota with no need to external inoculum and the absence of evident detrimental secondary effects. These advantages may be converted in economic advantages related to reduced filter volume and a more stable microbiota. Quantitative comparative studies and extended experimentation are now necessary to evaluate applicability of moss-based biofilters in aquaculture and aquaponics technologies.

## Figures and Tables

**Table 1:** α-diversity indices measured for the two moss species at the taxonomic level of phylum and family.

		Chao Index	Shannon Index	Berger-Parker
**Family**	*T. barbieri*	263.67	3.07	0.21
	*L. riparium*	281.93	3.03	0.18
**Phylum**	*T. barbieri*	39.0	1.56	0.51
	*L. riparium*	39.2	1.65	0.46

## Data Availability

The original contributions presented in this study are included in the article/Supplementary Materials. Further inquiries can be directed to the corresponding authors.

## Supplementary Materials

The following supporting information can be downloaded at: https://www.mdpi.com/article/10.3390/plants15030391/s1: Figure S1: Schematic diagram of the two compartments of the land-based aquaculture production macrosystem, one with traditional filter bed biofilter (green) and one with aquatic moss biofilter (blue). Table S1: Layers of filter-bed biofilter. Figure S2: Layers of the Classic filter-bed biofilter. (A) Detail of gravel paving; (B) Detail of porous ceramic cylinders paving; (C) Detail of expanded clay paving. Figure S3: Plants hosted in the biofilter. (A) overview of the tanks; (B) Detail of moss tanks hosting *L. riparium*; (C) Detail of tank hosting *Azolla filiculoides* showing decay after as little as one week; (D) Layout of the three filter bed biofilters (right) and the three aquatic moss biofilters (left). Description of the land-based assisted production macrosystem [59].

## Abbreviations

The following abbreviations are used in this manuscript:

RAS	Recirculating Aquaculture Systems
TAN	Total Ammonia Nitrogen
PCA	Plate Count Agar
SRG	Specific Growth Rate
