# Supplementary material for: Aquatic Moss Mats Are Alternative Biofilter Media for Aquaculture and Aquaponic Effluents Treating
Source: Plants (Basel). 2026 Jan 27;15(3):391. doi: 10.3390/plants15030391 (PMC12899261; doi:10.3390/plants15030391)
Supplement: Supplementary file 1 [file plants-15-00391-s001.zip › plants-4091704-supplementary.pdf]

SUPPLEMENTAL DATA

Aquatic Moss Mats Are Alternative Biofilter Media for Aquaculture and Aquaponic Effluents Treating

Irma Del Piano [1,†], Francesca Letizia [1,†], Matteo Calcagnile [1,†], Alessandro Sicuro [1,2], Laura Pecoraro [1,2], Elisa Quarta [1], Loredana Stabili [3,4], Tiziano Verri [1,2], Pietro Alifano [5], Fabrizio Barozzi [1,*] and Gian Pietro Di Sansebastiano [1,*]

Description of the land-based assisted production macrosystem

The system is located at the Urban Farming Lab of the University of Salento. It is divided into two distinct and independent compartments as described in the method section. The first compartment operates through a traditional biofiltration system with a filter bed, while the second relies on an aquatic moss biofilter. Each compartment consists of a fish production tank, a settling tank, three biofiltration tanks, two plant cultivation tanks, a process water storage and recirculation tank, a recirculation pump, a UV sterilizer, and a piping system that serves the entire facility. This configuration allows for effective management of the water cycle and biological filtration processes, enhancing water quality and ensuring the sustainability of the production system (Figure S1).

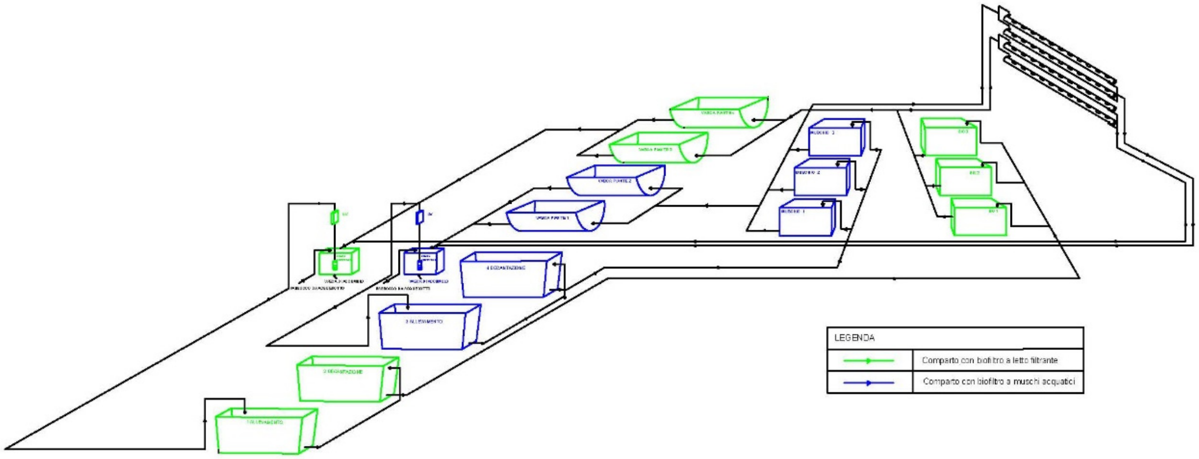

Figure S1. Schematic diagram of the two compartments of the land-based aquaculture production macrosystem, one with traditional filter bed biofilter (green) and one with aquatic moss biofilter (blue).

Classic filter-bed biofilter

The filter-bed biofilter used in the system consists of three tanks, each with a capacity of approximately 500 liters and dimensions of 96 cm x 84 cm x 64 cm, made of linear low-density polyethylene (LLDPE). The system operates in parallel, ensuring a fluid retention time of

approximately 2.5 hours. The design of the biofilter was based on the amount of organic matter introduced into the circuit daily in the form of feed, as well as the dimensions of the tanks dedicated to biofiltration.

The filter bed is composed of a porous material, known as a "carrier", which is sufficiently inert and characterized by a high specific surface area, referred to as Specific Surface Area (SSA) [m^2/m^3]. This parameter represents the ratio between the surface area available for bacterial colonization and the volume of the material. Such a feature is essential for promoting the growth of nitrifying bacteria, responsible for converting ammonia into nitrites and subsequently into nitrates.

To ensure effective biofiltration, the tanks are filled with three layers of inert materials, arranged from the bottom to the top. Specifically, the first layer consists of expanded clay for agricultural use (Agri Leca with neutral pH), followed by a second layer of porous ceramic cylinders (Sera Siporax Professional 15 mm), and finally, an upper layer of gravel. This configuration maximizes the surface available for nitrifying bacteria and optimizes the biological filtration process, as shown in Table S1 below.

Table S1: Layers of filter-bed biofilter

Material	Layer height (m)	Volume (m^3)	SSA (m^2/m^3)	Developed m^2
Gravel	0.07	0.06	150	8.40
Porous ceramic cylinders	0.05	0.04	1000	40.00
Expanded clay	0.13	0.10	200	20.80

As said, inside each tank of the traditional biofilter, it was planned to use a filter bed consisting of three components, in overlapping layers, and, in particular, arranged as follows starting from the top (Figure S2):

1) 10 cm of gravel of different grain size (2-15 mm) having both a large surface area per unit volume (SSA= 150 m^2/m^3) with the function of preventing the floating of the underlying layers thanks to its high density (Figure S2A).
2) 8 cm of porous ceramic cylinders (15 mm x 15 mm x 2,5 mm), having SSA = 310 m^2/m^3 (Figure S2B).
3) 10 cm of expanded clay (AgriLeca with neutral pH for agriculture), with a surface area index per volume of 200-250 m^2/m^3 (Figure S2C).

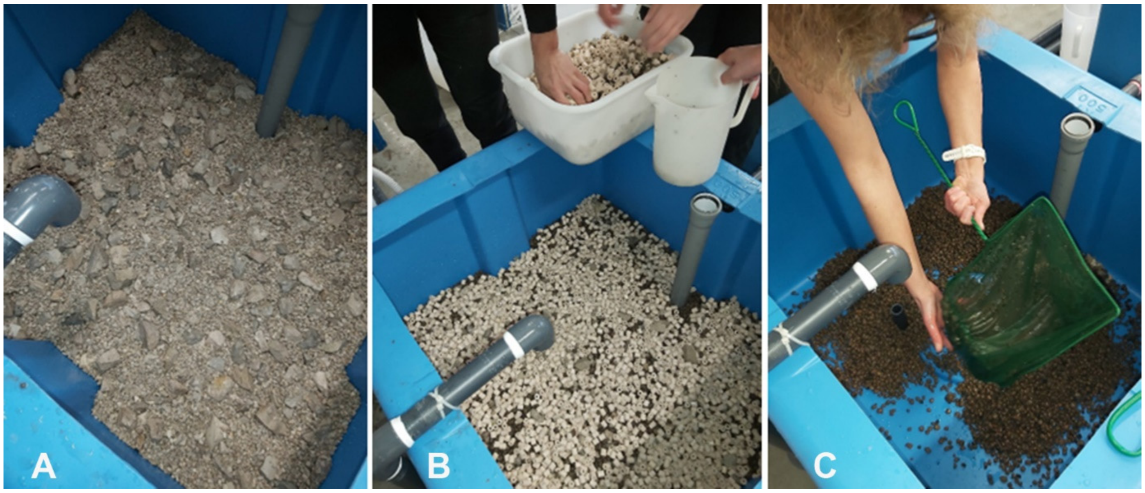

Figure S2: Layers of the Classic filter-bed biofilter. A) Detail of gravel paving; B) Detail of porous ceramic cylinders paving; C) Detail of expanded clay paving.

Inside the system, the three tanks that constitute the filter-bed biofilter develop a nitrifying bacterial colonization surface exceeding 200 m^2, with a volume of inert material of approx. 0.6 m^3. This configuration corresponds to a total SSA of about 350 m^2/m^3. Given that the ammonia removal rate by nitrifying bacteria is estimated at 0.57 g/m^2 per day and that the daily feed input is 200 g, resulting in the production of 7.5 g of ammonia, the operational surface required to fully nitrify this amount of feed is approximately 13.3 m^2 [59]. The biofilter was activated with commercial activator "Stability" of Seachem in a dose of 5 ml each 40 L of water pre-incubated for one week.

Aquatic moss biofilter

The aquatic moss biofilter, located downstream of the fish tanks, consists of five tanks arranged in series (Figure S3). The first three tanks submerged aquatic mosses, while the last two are entirely covered with small floating aquatic plants (Figure S3A). Specifically, 3 kg of *L. riparium* were introduced into the first three tanks of the biofilter (1 kg of live moss in each of the three tanks and the space occupied by the mass of moss corresponds to approximately 1/5 of it). Despite the absence of specific lighting, the moss successfully adapted to the biofilter environment, exhibiting vegetative growth rates consistent with expectations for such conditions (< 10% per month), without showing signs of necrosis or thallus degradation (Figure S3B).

Regarding the floating plant tanks, two species were introduced for short tests: *Lemna minor* and *Azolla filiculoides*. However, unlike the moss, these plants demonstrated more demanding physiological requirements. While *Lemna minor* adapted more easily to the biofilter environment, *Azolla filiculoides* experienced rapid decay (Figure S3C), leading to the formation of particulate matter, which could pose a potential issue for the filtration system. For these reasons, after one month only *L. minor* was left in the system. By operating the two biofilters in parallel, it was possible to directly assess and compare the efficiency of both systems (Figure S3D). Floating plants had a limited biomass and were tested for their growth performance, they were equally added to both circuits and had no relevant effects on biofiltration.

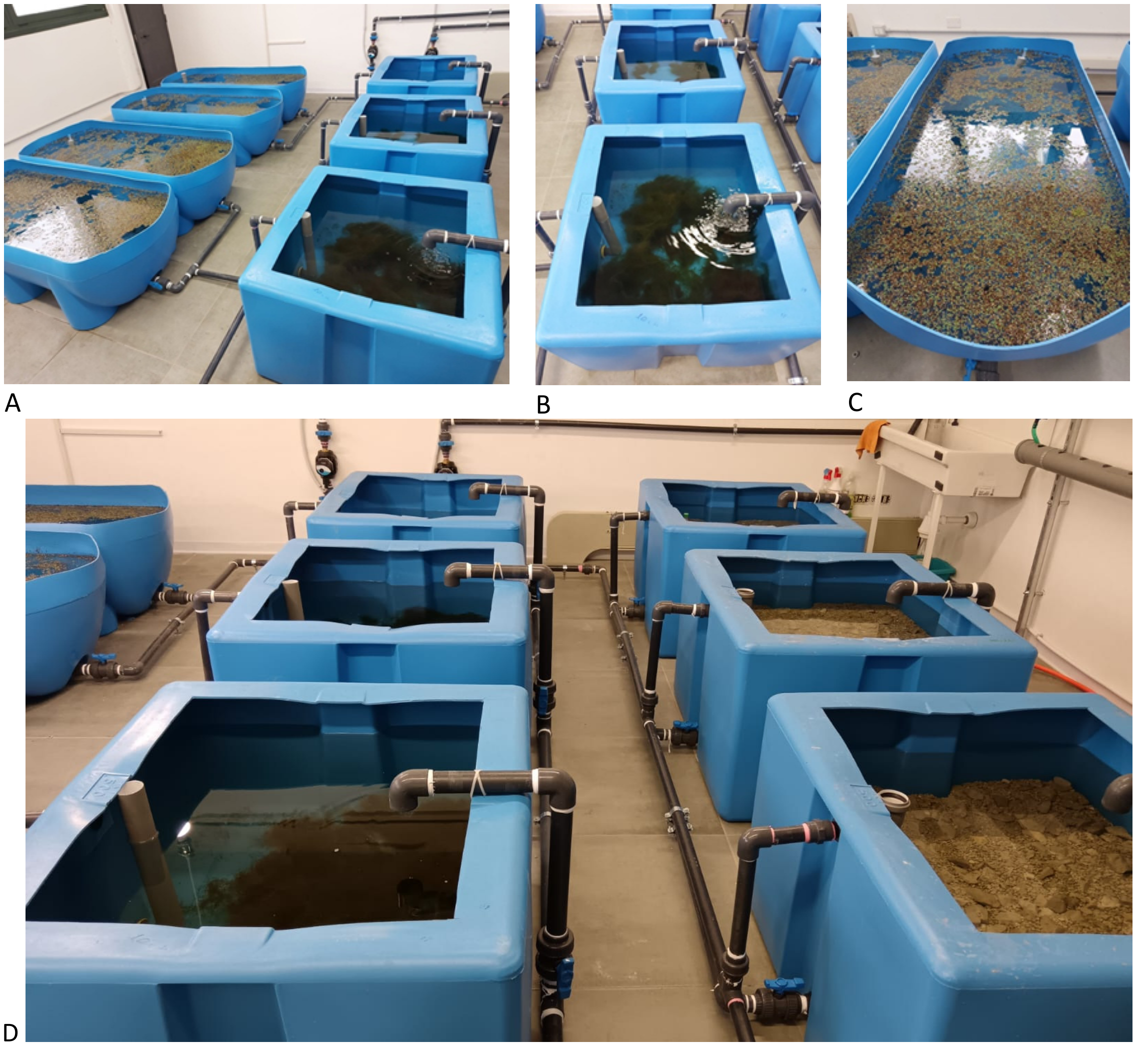

Figure S3: Plants hosted in the biofilter. A) overview of the tanks; B) Detail of moss tanks hosting *L. riparium*; C) Detail of tank hosting *Azolla filiculoides* showing decay after as little as one week; D) Layout of the three filter bed biofilters (right) and the three aquatic moss biofilters (left).
